# Preventive cardiology at an inflection point

**DOI:** 10.1016/j.ajpc.2026.101550

**Published:** 2026-03-12

**Authors:** Michael D. Shapiro

**Affiliations:** Center for Prevention of Cardiovascular Disease, Department of Cardiovascular Medicine, Wake Forest University School of Medicine, Medical Center Boulevard, Winston Salem 27157, NC, USA

Not long ago I saw a patient in clinic who illustrates a familiar challenge in cardiovascular disease (CVD) prevention. He was in his mid-fifties, overweight, with borderline hypertension, moderately elevated LDL-cholesterol, and early insulin resistance. His coronary artery calcium score was 58. He had no history of cardiovascular events nor symptoms suggestive of CVD. By most conventional standards he was not yet “sick.” Yet anyone who spends time in preventive cardiology recognizes the trajectory. Left alone, the risk factors slowly compound and the first clinical event often arrives years later. The question we face with patients like this is straightforward. We know a great deal about the biology of cardiometabolic disease and about how to reduce risk. The challenge is whether our clinical systems can act on that knowledge early enough to matter.

Within a short span of time our field is releasing a series of major prevention guidelines that reshape how we think about cardiometabolic risk. The updated Multisociety Hypertension guidelines were just published and the new 2026 Dyslipidemia guideline has just been released. Additional national guidelines addressing cardiovascular kidney metabolic disease and primary prevention of CVD are close behind. Each represents years of work and reflects important advances in the science of prevention. Our field is being reorganized in real time.

Blood pressure, lipids, metabolic disease, kidney dysfunction, obesity, and atherosclerosis are increasingly understood not as isolated problems but as interconnected expressions of a single disease continuum. The guidelines now emerging from our major societies reflect that reality. They push us toward earlier identification of risk, more comprehensive treatment strategies, and a broader view of cardiometabolic health. Conceptually, this shift is long overdue.

In practice, the publication of new guidelines does not guarantee that patients will benefit from them any time soon. Evidence can accumulate quickly while clinical practice evolves far more slowly. In CVD prevention this gap is visible every day. Large numbers of high risk patients still do not receive guideline directed lipid lowering therapy. Blood pressure control remains suboptimal across much of the population. Therapies that have been available for years are used inconsistently or not at all. At the same time clinicians responsible for preventive care face an expanding set of recommendations layered onto already crowded clinical workflows.

The problem is that the systems responsible for delivering prevention were never built to absorb this level of complexity. Guidelines assume a clinical infrastructure that often does not exist. They assume time for careful risk discussion and shared decision making. They assume access to recommended therapies. They assume decision support tools that help clinicians integrate multiple risk frameworks. They assume team-based care models that distribute the work of prevention across physicians, advanced practice providers, pharmacists, nurses, and dietitians.

Many clinicians operate in environments where those assumptions do not hold. Primary care physicians manage enormous patient panels. Electronic health record alerts accumulate faster than they can be processed. Quality metrics often lag behind evolving evidence. Formularies and insurance policies sometimes limit access to therapies that guidelines recommend. While extremely challenging, this also provides an enormous opportunity.

The simultaneous emergence of hypertension, dyslipidemia, CKM, and primary prevention guidelines creates a rare moment for alignment across the field. Rather than treating these documents as separate sets of recommendations, we should recognize that they describe different dimensions of the same clinical problem. Cardiometabolic disease develops over decades through the interaction of multiple biological pathways. Our approach to prevention must be equally integrated. If these frameworks are implemented separately, each filtered through a different specialty lens, fragmentation will persist. If they are implemented together, they offer the possibility of a far more coherent model of preventive care.

This is where we can lead. Our discipline sits at the intersection of several traditionally separate domains. Lipidology, hypertension management, metabolic disease, obesity medicine, imaging, and risk prediction all converge in the daily work of preventive cardiology clinics. Preventive cardiologists think in terms of lifetime risk, disease trajectory, and the interaction of multiple pathways that lead to cardiovascular events. That perspective is exactly what the new generation of guidelines demands.

However, if these recommendations are going to change patient outcomes, we must build the infrastructure that allows clinicians to use them effectively. That work extends well beyond the writing of guidelines. It includes training clinicians who specialize in prevention, developing practical clinical tools that translate complex recommendations into real decisions, and working with health systems so preventive care is supported rather than obstructed.

The American Society for Preventive Cardiology has increasingly focused on the practical challenges of delivering prevention in real clinical environments. Educational initiatives, training programs, and collaborative research efforts are all part of a broader effort to strengthen the field’s capacity to implement evidence-based care. Evidence changes practice only when clinicians have the knowledge, the tools, and the support necessary to act on it.

The convergence of new guidelines should therefore be seen as an opportunity to reconsider how preventive cardiology is delivered. We now have stronger evidence than ever about how to reduce cardiovascular risk. We have therapies that target multiple biological pathways. We have conceptual frameworks that integrate cardiovascular, metabolic, and kidney disease into a unified model of cardiometabolic health. What remains uncertain is whether our clinical systems will evolve quickly enough to translate that knowledge into everyday care.

Patients like the man I saw in clinic that day are not rare. They represent millions of individuals moving quietly along the path toward cardiovascular disease. The science to change that trajectory already exists. The challenge before us is to ensure that the systems of care are capable of acting on it. Preventive cardiology stands at an inflection point.Our responsibility is to ensure that these advances do not remain confined to journals and conference presentations. They must become part of routine clinical practice. The next era of CVD prevention will not be defined only by the guidelines we write, but by how effectively we bring those guidelines to life for the patients who depend on them.

Michael D. Shapiro

President, American Society for Preventive Cardiology

## CRediT authorship contribution statement

**Michael D. Shapiro:** Conceptualization, Writing – original draft, Writing – review & editing.

## Declaration of competing interest

The authors declare the following financial interests/personal relationships which may be considered as potential competing interests:

Michael D. Shapiro reports a relationship with Novartis that includes: consulting or advisory. Michael D. Shapiro reports a relationship with Amgen Inc that includes: consulting or advisory. Michael D. Shapiro reports a relationship with Merck & Co Inc that includes: consulting or advisory. Michael D. Shapiro reports a relationship with Alnylam Pharmaceuticals Inc that includes: consulting or advisory. Michael D. Shapiro reports a relationship with AstraZeneca that includes: consulting or advisory. If there are other authors, they declare that they have no known competing financial interests or personal relationships that could have appeared to influence the work reported in this paper.

